# Supplementary material for: Development of a computer-aided design software for the quantitative evaluation of aesthetic damage
Source: PLoS One. 2019 Dec 18;14(12):e0226322. doi: 10.1371/journal.pone.0226322 (PMC6919621; doi:10.1371/journal.pone.0226322)
Supplement: S1 Code site — 1. (ZIP) [file pone.0226322.s001.zip › [EESC_jr][Projeto]Relatorio_07_21/reports/templates_zip/gentelella-master/vendors/morris.js/examples/dst.html]

# Daylight-savings time

```
// This crosses a DST boundary in the UK.
Morris.Area({
  element: 'graph',
  data: [
    {x: '2013-03-30 22:00:00', y: 3, z: 3},
    {x: '2013-03-31 00:00:00', y: 2, z: 0},
    {x: '2013-03-31 02:00:00', y: 0, z: 2},
    {x: '2013-03-31 04:00:00', y: 4, z: 4}
  ],
  xkey: 'x',
  ykeys: ['y', 'z'],
  labels: ['Y', 'Z']
});
```
